# 

**Published:** 1803-11-01

**Authors:** 


### TO CORRESPONDENTS.

Richerand's Elements of Physiology will be noticed in our next number.

Communications are received from Mr. Nayler, Mr. Roche, Mr. Morgan, Mr. Dunn, Mr. Blair, and from the Continent.

